# Health services costs for cancer care in Australia: Estimates from the 45 and Up Study

**DOI:** 10.1371/journal.pone.0201552

**Published:** 2018-07-30

**Authors:** David E. Goldsbury, Sarsha Yap, Marianne F. Weber, Lennert Veerman, Nicole Rankin, Emily Banks, Karen Canfell, Dianne L. O’Connell

**Affiliations:** 1 Cancer Research Division, Cancer Council NSW, Sydney, Australia; 2 Sydney School of Public Health, University of Sydney, Sydney, Australia; 3 School of Medicine, Griffith University, Southport, Queensland, Australia; 4 Sydney Catalyst, NHMRC Clinical Trials Centre, Chris O’Brien Lifehouse Building, Camperdown, New South Wales, Australia; 5 National Centre for Epidemiology and Population Health, Australian National University, Canberra, Australian Capital Territory, Australia; 6 Prince of Wales Clinical School, UNSW Medicine, Sydney, New South Wales, Australia; 7 School of Medicine and Public Health, University of Newcastle, Newcastle, New South Wales, Australia; Centro per lo Studio e la Prevenzione Oncologica, ITALY

## Abstract

**Background:**

Cancer care represents a substantial and rapidly rising healthcare cost in Australia. Our aim was to provide accurate population-based estimates of the health services cost of cancer care using large-scale linked patient-level data.

**Methods:**

We analysed data for incident cancers diagnosed 2006–2010 and followed to 2014 among 266,793 eligible participants in the 45 and Up Study. Health system costs included Medicare and pharmaceutical claims, inpatient hospital episodes and emergency department presentations. Costs for cancer cases and matched cancer-free controls were compared, to estimate monthly/annual excess costs of cancer care by cancer type, before and after diagnosis and by phase of care (initial, continuing, terminal). Total costs incurred in 2013 were also estimated for all people diagnosed in Australia 2009–2013.

**Results:**

7624 participants diagnosed with cancer were matched with up to three controls. The mean excess cost of care per case was AUD$1,622 for the year before diagnosis, $33,944 for the first year post-diagnosis and $8,796 for the second year post-diagnosis, with considerable variation by cancer type. Mean annual cost after the initial treatment phase was $4,474/case and the mean cost for the last year of life was $49,733/case. In 2013 the cost for cancers among people in Australia diagnosed during 2009–2013 was ~$6.3billion (0.4% of Gross Domestic Product; $272 per capita), with the largest costs for colorectal cancer ($1.1billion), breast cancer ($0.8billion), lung cancer ($0.6billion) and prostate cancer ($0.5billion).

**Conclusions:**

The cost of cancer care is substantial and varies by cancer type and time since diagnosis. These findings emphasise the economic importance of effective primary and secondary cancer prevention strategies.

## Introduction

Cancer care accounts for a substantial proportion of healthcare expenditure. The number of people diagnosed with cancer in Australia is rising, due to an ageing and increasing population, lifestyle and environmental factors [1]. Advances in cancer care, including new technologies and targeted therapies, are increasing the case-specific costs of care [2–4]. Cancer survival is also increasing, with more people living after a cancer diagnosis who require continuing care—approximately 1 million Australians in 2012 [5]. Hence, the cost of cancer care in Australia is increasing rapidly. High quality data on current cancer care costs are needed to prioritise future healthcare funding, assess the cost-effectiveness of potential interventions relating to cancer control, and plan for future costs.

The Australian healthcare system includes government-funded universal coverage of many medical costs, along with further coverage via private health insurance or self-funding. Healthcare costs directly attributable to cancer were an estimated $4.5billion Australian dollars (AUD) in 2009, accounting for 4% of all government expenditure in health [6]. This represented a 25% increase in total expenditure from 2005, which was a similar increase from the 2001 estimate. Another Australian report for the state of New South Wales (NSW; 32% of Australia’s population) estimated $1.1billion for lifetime health system costs for people diagnosed with cancer in 2005 [7]. Both reports used an approach that only covered costs of care specifically identifiable as being for cancer treatment, and excluded costs for care not clearly indicated for cancer alone, such as general practitioner consultations. Other Australian studies have reported on expenditure related to national cancer-specific prevention programs, such as cervical cancer screening costs [8].

Australian cancer costing studies using individual-level information have focused on specific cancer types in specific geographical locations, including prostate, lung and skin cancers in NSW [9–11] and colorectal cancer in Victoria [12]. The latter study highlighted the impact of specific new drugs on treatment costs for advanced colorectal cancer, estimated to add over AUD$10,000 for each case using bevacizumab or cetuximab. Other NSW studies have focused on chemotherapy costs [13] or end-of-life care [14, 15]. A recent series of papers focused on the cost of cancer to patients, has highlighted the relatively high out-of-pocket costs and associated ‘financial toxicity’ for Australian cancer patients and their carers [16–18], along with the complexity of funding structures and methods used to measure costs [19, 20].

Previous studies in the UK, the US and New Zealand have reported on the excess health system costs of care for cancer patients relative to cancer-free controls for several cancer types and over different phases of care, providing a valuable resource for expenditure priority-setting and economic evaluations of potential interventions [21–23]. To our knowledge, there are no equivalent Australian studies encompassing all cancer types.

The aim, therefore, of this study was to estimate the costs of cancer care for all cancers in Australia using patient-level data for a large population-based cohort. This includes health system costs by cancer type and overall, based on several time periods relative to diagnosis and death. We included the full range of health system costs of cancer care, from pre-diagnosis, through initial treatment, continuing care and end-of-life care. This will provide a detailed baseline with which to compare future costs and facilitate assessment of the cost-effectiveness of potential new interventions in cancer control.

## Materials and methods

### Data sources

We used data from The Sax Institute’s 45 and Up Study, a longitudinal study of over 266,000 people in NSW aged > = 45 years. The study methods and cohort have been described in detail previously [24]. Briefly, potential participants were a random sample from the Medicare enrolment database held by the Department of Human Services (formerly Medicare Australia), which provides near complete coverage of the population. People aged > = 80 years and those living in regional and remote areas were oversampled by a factor of two. Participants completed a baseline postal questionnaire in 2006–2009 and consented to linkage to their routinely collected health information. This includes reimbursements for (1) subsidised prescription medicines in the Pharmaceutical Benefits Scheme (PBS), (2) subsidised outpatient and medical services and some in-hospital procedures covered by the Medicare Benefits Schedule (MBS), (3) inpatient care in public and private hospitals from the Admitted Patient Data Collection (APDC) and (4) emergency presentations from the Emergency Department Data Collection (EDDC). The study period was 1 January 2005 to 30 June 2014, during which time data from all sources were available (Figure A in S1 File). Linkage to the (5) NSW Cancer Registry (NSWCR) enabled identification of all incident and prevalent cancers for the cohort (excluding non-melanoma skin cancer), and (6) death records were obtained from the NSW Registry of Births, Deaths and Marriages (RBDM). Individual records were linked to health databases (1) and (2) by the Sax Institute using a unique identifier that was provided to the Department of Human Services, while individual records in databases (3) to (6) were probabilistically linked by the Centre for Health Record Linkage in NSW using a best practice approach to linkage while preserving privacy [25].

### Study sample

**Fig 1:**
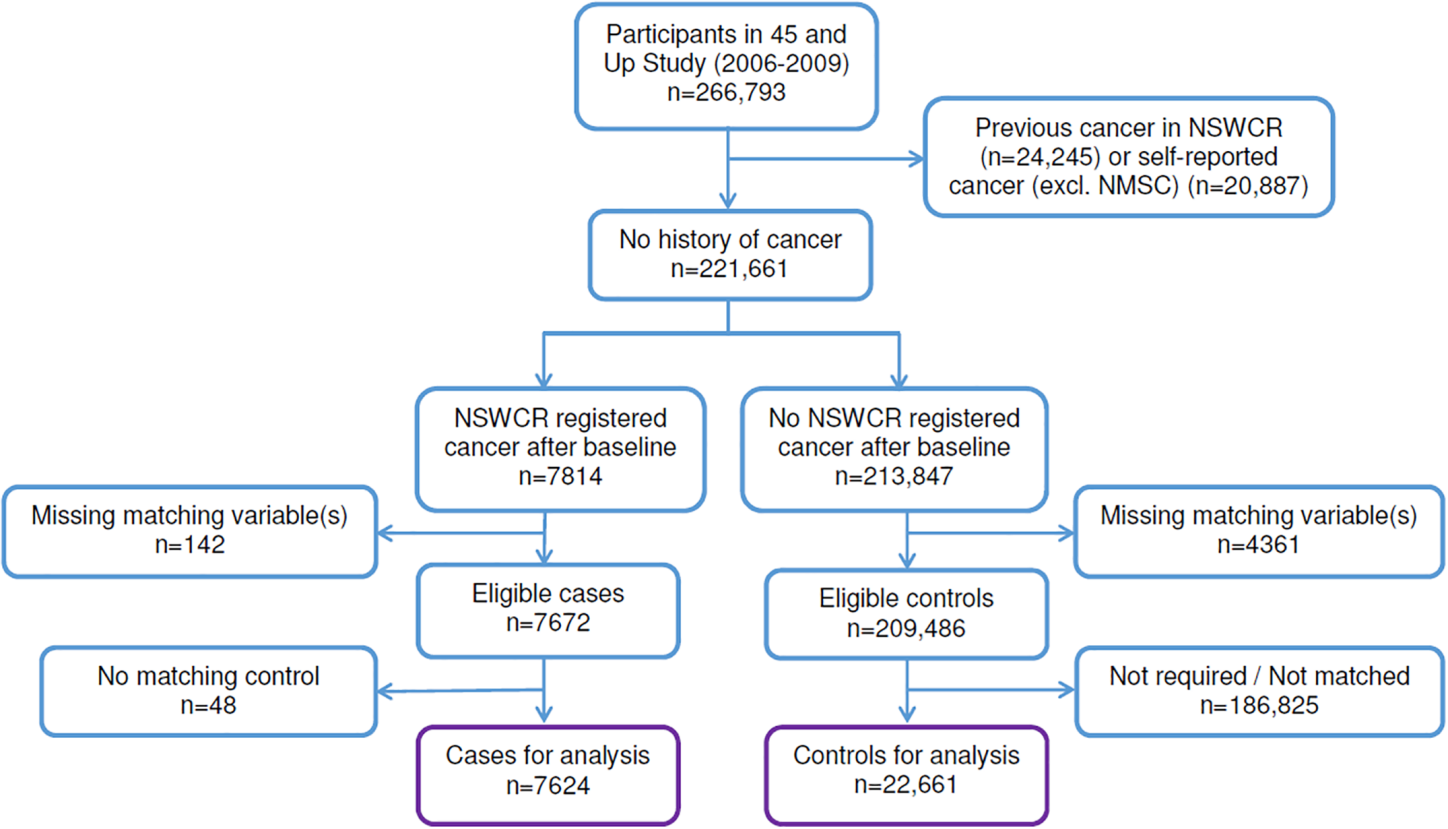
Cohort selection flow diagram. NMSC: Non-melanoma skin cancer; NSWCR: New South Wales Cancer Registry. Matching variables were age (±5 years), sex, Local Government Area of residence and smoking history (never or quit >15 years, recent quitter within 15 years, current smoker).

People included in this analysis were 45 and Up Study participants with a NSWCR-registered cancer diagnosis during 2006–2010 that occurred after study recruitment (“incident cases”). We excluded people with a cancer diagnosis recorded in the NSWCR prior to recruitment and those with a self-reported history of cancer (excluding non-melanoma skin cancer) (Fig 1). Cases were matched to cancer-free controls who had no record of cancer and who did not die prior to the diagnosis date of their potentially matched case. There was no restriction on controls who died after the diagnosis date, so that the results are not biased by comparing the costs for the cancer cases with those for a healthy control group. Up to three controls were matched to each case (without replacement) by age (±5 years), sex (female, male), Local Government Area of residence (approximately 150 areas in NSW) and smoking history (never or quit >15 years, recent quitter within 15 years, current smoker). People with missing responses for any of the characteristics used in matching were excluded, as were 50 participants with irreconcilable information in their linked records (e.g. multiple hospital admissions after recorded date of death) or who were aged <45 years at baseline.

### Definitions and calculations of costs

#### Cost data

Costs were based on individuals’ inpatient hospital episodes, emergency department (ED) presentations, subsidised prescription medicines captured in the PBS and subsidised medical services captured in the MBS. The PBS includes medicines for the prevention or treatment of significant medical conditions, with subsidised drugs approved by the government after first being approved by the Therapeutic Goods Administration (similar to the US Food and Drug Administration) and then undergoing an assessment of their cost-effectiveness by the Pharmaceutical Benefits Advisory Committee [26]. All cases had at least 3.5 years of follow-up after diagnosis (median 5 years). Costs were estimated for several time periods relative to the diagnosis date; if the time period of interest extended to dates outside of the study period then the matched case-control ‘group’ were not included in the estimates for that specific period (e.g. if diagnosed in 2010 then 5 years post-diagnosis is after the end of the study period). All items and procedures were included and not restricted to health care deemed to be cancer-specific.

For this study we focused on direct health system (government-funded) costs. The dates of supply of prescription medicines and medical services from the PBS and MBS respectively were used to assign the costs to the relevant time period. Inpatient hospital costs were derived by linking the Australian Refined Diagnostic Related Group code for each hospitalisation to the 2010 National Hospital Cost Data Collection (NHCDC) average admission cost [27]. Hospitalisations could span multiple time periods, so costs were apportioned across time periods according to the number of days out of the total length of stay. ED costs were assigned according to triage category and discharge status as described in the NHCDC.

#### Statistical analysis

The excess costs due to cancer were calculated for each case by taking their costs and subtracting the average costs for their matched controls. We calculated annual and monthly incidence costs, along with costs by phase of care (initial, continuing, terminal) and prevalence costs. Means of excess costs were calculated and converted to 2013 Australian dollars using the Australian Health Index [28]. The proportions of excess costs contributed by each source (inpatient hospitalisations, prescription medicines, other services subsidised by the MBS, and ED presentations) were calculated. Medians and inter-quartile ranges for excess costs are reported in the Supporting Information.

Costs were calculated for the ten most common cancer types in Australia and all other cancers combined. Specific cancer types were identified using the NSWCR topography code, based on the International Classification of Diseases, Tenth Revision, Australian Modification (ICD-10-AM; see Supporting Information for codes). Cases were also classified by summary disease stage at diagnosis (localised, regional, distant metastases, unknown). We compared the distribution of cancer types in our cohort with the population-wide distribution of incident cancers in all of Australia in 2013 [5], as well as with the distribution of incident cancers by stage in NSW in 2008–2012 [29] (both were the most recent data available), weighting the study cohort to match these distributions. Analysis was carried out using SAS v9.4 (SAS Institute Inc., NC, USA).

#### Costs for incident cancers

The annual costs for incident cancers were calculated for each participant for each 12-month period around the case’s month of diagnosis, from 2 years pre-diagnosis to 5 years post-diagnosis. Only month and year of diagnosis were available so the cost for the first 12 months “after” diagnosis started on the first day of the month of diagnosis. Monthly costs were also calculated for each calendar month relative to the month of diagnosis. For each of these annual and monthly estimates, if a case and/or their matched control died then the included costs were censored at the earliest date of death and the case-control pair were excluded from estimates for subsequent years and months. This means, for example, that the denominator for costs for >1–2 years post-diagnosis is all cases who survived at least one year after diagnosis and who had at least one matched control still alive one year after the diagnosis date. We also calculated costs for each month at the end of life, relative to the death date. If a control died before their matched case then the end-of-life costs were censored at the control’s death date and the case-control pair was excluded from estimates for the subsequent months. For example, if the control died 4.5 months before their matched case then this case-control pair was excluded from estimates for the final four months of end-of-life costs.

#### Costs by phase of care

Costs were also grouped into three phases of care: the initial phase, the continuing phase and the terminal phase. The phases for each matched group were assigned based on the case’s diagnosis date, death date and the end of follow-up. For estimates corresponding to each phase, if a control died before their matched case then the included costs were censored at the control’s date of death and the case-control pair was excluded from estimates for subsequent phases.

Initial phase: If the case survived at least two years then the first year after diagnosis was designated the initial phase. If the case survived >1 year but <2 years after diagnosis then the initial phase was the period from diagnosis until the start of the 12-month terminal phase.

Continuing phase: If the case survived >2 years after diagnosis then the period between the end of the initial phase and the start of the terminal phase, or 31 December 2013 if the case did not die, was designated the continuing phase. The costs for this phase were calculated as an annual rate for each individual according to the length of their continuing phase. To avoid incorrect over-inflation, if the continuing phase was <3 months then the case-control group were not included in this phase (2% of groups).

Terminal phase: For cases who died before July 2014, the final year up to and including the death date was designated the terminal phase. If the case survived <1 year after diagnosis then the terminal phase started at the diagnosis date and the case had no initial phase.

#### Prevalence costs

Costs incurred during 2013 for people living up to 5 years after a cancer diagnosis were estimated using the numbers of people diagnosed with cancer in Australia during 2009–2013 [5] and survival rates for each year after diagnosis by cancer type [30]. The mean cost for the year after diagnosis was applied to the numbers diagnosed in Australia in 2013 by cancer type. The 1-year survival rate by cancer type was applied to the numbers diagnosed in 2012 to give the expected number surviving to 2013, and this was multiplied by the mean cost by cancer type for the second year after diagnosis. The same was done for the third year after diagnosis using incidence in 2011 and 2-year survival, for the fourth year after diagnosis using incidence in 2010 and 3-year survival, and for the fifth year after diagnosis using incidence in 2009 and 4-year survival. The totals for the five years were combined to obtain an overall prevalence cost for 2013.

#### Ethical approval

The University of New South Wales Human Research Ethics Committee approved the conduct of the 45 and Up Study and the NSW Population and Health Services Research Ethics Committee (approval number 2014/08/551) approved this study.

## Results

### Study sample

Of the 266,793 eligible participants in the 45 and Up Study there were 7,672 eligible cases diagnosed with cancer between baseline and December 2010 (Fig 1). At least one matching control was available for 7,624 cases (99.4%), with three matching controls for 7,473 (97.4%), two matching controls for 91 (1.2%) and one matching control for 60 (0.8%), giving the final study sample of 7,624 cases and 22,661 controls. The mean age of the cases at diagnosis was 69 years, 60% were men, 53% were from major cities, 12% were from rural areas, 7% were smokers at baseline and 14% had quit smoking within the previous 15 years.

**Fig 2:**
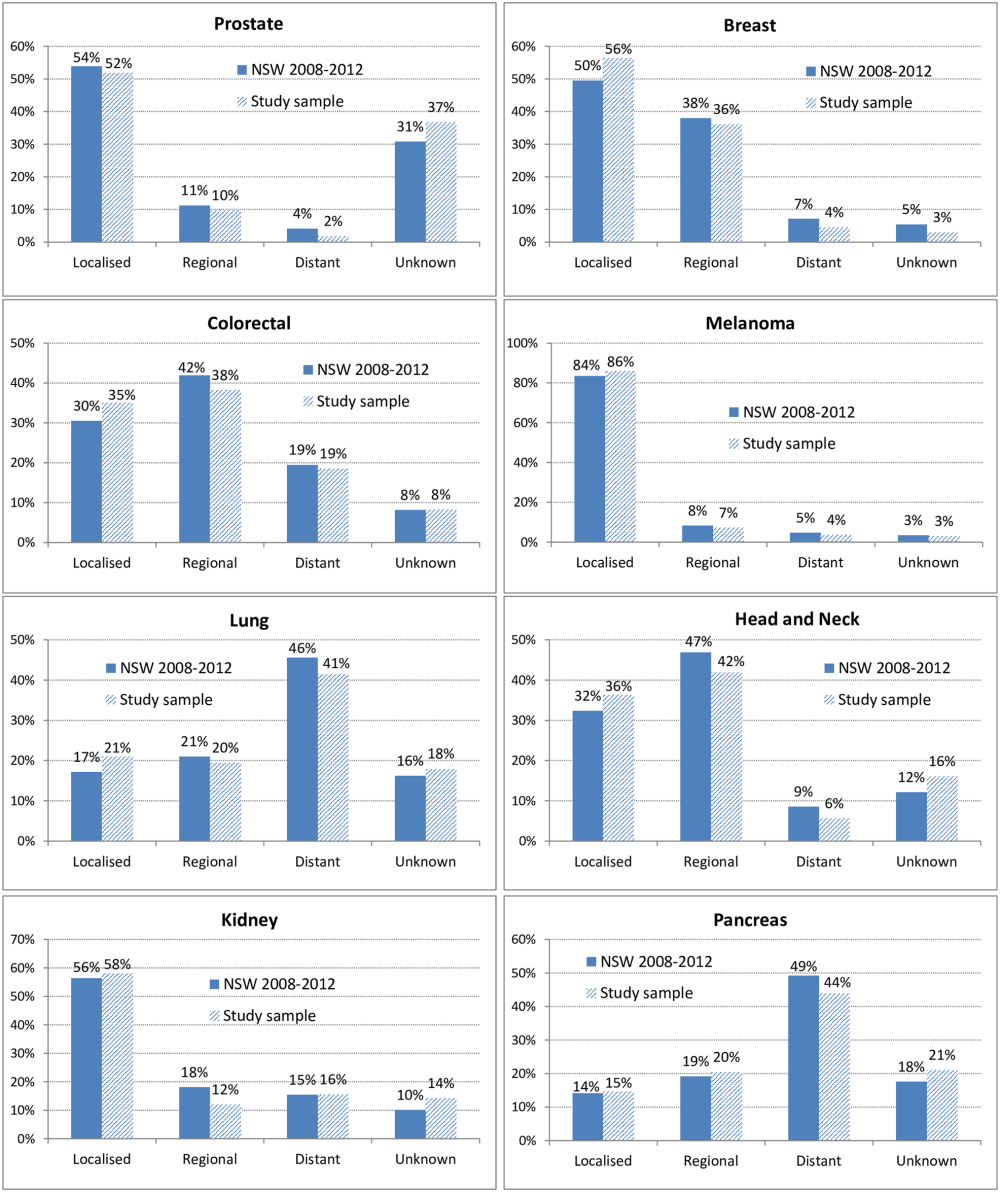
Distribution of cases included for analysis (diagnosed 2006–2010) by spread of disease at diagnosis and cancer type, compared with all cases in New South Wales (NSW) diagnosed 2008–2012. Results not shown for Non-Hodgkin lymphoma or leukaemia, ~90% recorded as “Unknown” for both.

The distribution of cancer types was reasonably similar to that for all incident cancers in Australia in 2013, with the exception of prostate cancer which accounted for 25% of the study sample compared with 15% of all Australian cases (Table A in S1 File). The distribution of stage of disease for each of the 10 most common cancer types was reasonably similar to that for all incident cancers in NSW in 2008–2012 (Fig 2). Thirty-seven percent of the cancer cases in the study sample were diagnosed in 2010, 35% were diagnosed in 2009 and 19% in 2008; at June 2014 81% had at least 4 years of post-diagnosis cost data available. Fifteen percent of cases died within one year of diagnosis and 28% died within four years; 6% of controls died within four years. Survival varied by cancer type, for example 52% of lung cancer cases died within one year of diagnosis compared with 2% of breast and prostate cancer cases.

### Costs of incident cancers

Costs varied by cancer type and time since diagnosis, with the annual costs dominated by those occurring in the first year after diagnosis (including the month of diagnosis) (Table 1). Among the most common cancer types, the mean cost for the first year after diagnosis ranged from $7,110 per case for melanoma to $48,570 for colorectal cancer. Weighted to the distribution of cancer types in Australia, the mean cost was $1,622 per case for the year prior to diagnosis, $33,944 for the first year after diagnosis and $8,796 for the second year after diagnosis.

**Table 1:** Annual mean excess costs by cancer type for incident cancers diagnosed 2006–2010, in 2013 Australian dollars.

Cancer type^1^	>1–2 years pre-diag.		>0–1 year pre-diag.		0–1 year post-diag.		>1–2 years post-diag.		>2–3 years post-diag.		>3–4 years post-diag.		>4–5 years post-diag.	
	n	Mean ($)	n	Mean ($)	n	Mean ($)	n	Mean ($)	n	Mean ($)	n	Mean ($)	n	Mean ($)
Prostate	1,860	-153	1,944	-275	1,944	18,159	1,857	2,830	1,765	2,314	1,385	2,157	759	1,678
Breast	980	-299	1,010	-576	1,010	36,948	986	7,619	956	3,038	748	3,132	408	2,473
Colorectal	926	15	967	1,545	967	48,570	816	10,926	720	7,381	515	6,509	274	4,525
Melanoma	852	582	891	565	891	7,110	838	2,260	784	2,792	604	2,506	327	1,784
Lung	495	-8	523	2,263	523	38,059	247	17,863	168	12,903	102	6,563	46	4,021
NHL	251	-177	261	2,102	261	46,286	223	11,485	204	6,997	158	4,741	75	2,305
Head & neck	139	341	143	1,290	143	42,305	121	5,907	109	-1,086	84	2,026	42	1,687
Leukaemia	140	-219	143	1,137	143	47,649	94	26,629	76	18,365	63	14,701	33	18,142
Kidney	137	267	141	4,207	141	35,882	119	9,020	103	9,841	81	7,953	48	9,598
Pancreas	151	664	157	3,705	157	44,903	50	22,217	25	11,412	13	308	7	21,477
Other	1,396	1,129	1,444	3,631	1,444	38,828	1,011	12,098	828	8,781	570	6,438	289	5,237
**Overall**^2^	**7,327**	**314**	**7,624**	**1,622**	**7,624**	**33,944**	**6,362**	**8,796**	**5,738**	**5,624**	**4,323**	**4,454**	**2,308**	**3,643**

n: number of cases alive at the start of the time period who had sufficient follow-up to be included; NHL: Non-Hodgkin lymphoma.

^1^ Weighted to the New South Wales disease stage distribution within each cancer type.

^2^ Weighted to the distribution of cancer types in Australia in 2013 and the New South Wales disease stage distribution.

The annual excess costs for incident cases were dominated by inpatient hospital costs, comprising 68% of costs for the first year after diagnosis, while 18% of costs were for other medical services subsidised by the MBS, 13% were for prescription medicines and 1% for ED presentations. The proportion of costs attributed to prescribed medicines increased to ~25% in the subsequent years with the proportion of hospital inpatient costs reducing to ~55%. Health system costs for controls over the whole study period were mainly for inpatient care (53%), followed by medical services subsidised by the MBS (27%), prescription medicines (17%) and ED presentations (3%).

The monthly excess costs for cases were highest in the month of diagnosis ($8386) and the months that followed (Figure B in S1 File). The proportion of costs for medical services covered by the MBS increased leading up to diagnosis, accounting for 36–41% of costs in the 3 months pre-diagnosis before falling to 15–19% per month from diagnosis onwards. Inpatient hospital costs accounted for ~80% of costs for the month of diagnosis and the month after diagnosis, reducing to ~50% after 12 months, with a corresponding increase in the proportion of costs attributed to prescription medicines.

For cases who were recorded as having died, there was a gradual increase in costs from 12 months prior to death then a rapid increase in the final 2–3 months, averaging $16,111 during the final month of life. This distribution of costs was similar regardless of survival time, apart from those surviving <1 year after diagnosis, who had slightly higher costs (Figure C in S1 File). The breakdown of costs for >5–12 months prior to death was consistently ~65% for inpatient hospital costs, 19% for prescription medicines and 15% for other medical services covered by the MBS. During the final month of life 89% of costs were for inpatient hospital care and there was a record of dying in hospital for 66% of cases.

### Costs by phase of care

Overall the mean cost for the initial treatment phase was $28,719 per case. The lowest cost by cancer type was $5,372 per melanoma case and the highest exceeded $40,000 for colorectal cancer and non-Hodgkin lymphoma (NHL) (Table 2). Melanoma cases had low prescription medicine costs, accounting for <1% of costs, whereas prescription medicines (e.g. rituximab) accounted for 37% of costs for NHL cases. Colorectal cancer costs were dominated by inpatient care, with 80% of cases having bowel surgery recorded during the initial phase. The initial costs were slightly higher for those with shorter survival time after diagnosis and lowest for those who survived >5 years.

**Table 2:** Mean excess costs for incident cancers diagnosed 2006–2010 by phase of care, cancer type and source of costs, in 2013 Australian dollars.

Cancer type^1^	Initial phase				Continuing phase (per year)				Terminal phase			
	Mean cost	% Hosp.-based	% MBS	% PBS	Mean cost	% Hosp.-based	% MBS	% PBS	Mean cost	% Hosp.-based	% MBS	% PBS
Prostate	$17,412	62%	33%	5%	$1,555	28%	47%	25%	$40,729	82%	9%	9%
Breast	$36,448	44%	25%	31%	$4,023	20%	29%	51%	$38,619	65%	13%	22%
Colorectal	$44,016	77%	13%	11%	$5,998	49%	20%	30%	$59,270	77%	9%	14%
Melanoma	$5,372	63%	36%	0%	$1,168	50%	43%	7%	$41,974	83%	13%	4%
Lung	$29,337	58%	23%	19%	$6,493	6%	26%	69%	$43,368	76%	11%	13%
NHL	$40,822	47%	16%	37%	$6,221	28%	21%	51%	$65,158	81%	9%	10%
Head & neck	$32,850	69%	29%	2%	-$1,796	N/A	N/A	N/A	$66,152	89%	9%	1%
Leukaemia	$36,176	56%	17%	26%	$15,070	22%	11%	68%	$63,462	85%	12%	3%
Kidney	$31,537	81%	15%	4%	$7,409	44%	20%	36%	$54,694	76%	10%	14%
Pancreas	$37,447	70%	20%	10%	$14,352	57%	15%	28%	$43,163	84%	10%	7%
Other	$32,042	74%	18%	9%	$7,133	60%	21%	19%	$50,327	80%	10%	10%
**Overall**^2^	**$28,719**	**64%**	**21%**	**15%**	**$4,474**	**40%**	**25%**	**35%**	**$49,733**	**79%**	**10%**	**11%**

Hosp.-based: Hospital-based services, combining Admitted Patient Data Collection and Emergency Department costs, the latter accounts for 0–3% of costs for each phase; MBS: Medicare Benefits Schedule; NHL: Non-Hodgkin lymphoma; N/A: Not applicable due to negative excess costs; PBS: Pharmaceutical Benefits Scheme.

^1^ Weighted to the New South Wales disease stage distribution within each cancer type.

^2^ Weighted to the distribution of cancer types in Australia in 2013 and the New South Wales disease stage distribution.

The mean annual cost for the continuing phase was $4,474 per case, with costs ranging from $1,168 per year for melanoma and prostate cancer to ~$15,000 per year for leukaemia and pancreatic cancer (Table 2). During the continuing phase 40% of costs were for inpatient care, 35% for prescription medicines and 25% for other medical services covered by the MBS. The mean excess cost during the continuing phase for head and neck cancers was negative, however this was caused by a small number of matched controls with extremely high costs (see Supporting Information notes). The higher costs for leukaemia were driven by prescription medicines (e.g. imatinib), accounting for 68% of costs in this phase. For pancreatic cancer the higher costs were generally for inpatient care, although this was based on relatively few cases.

The mean cost for the terminal phase was $49,733 per case, ranging from ~$40,000 for breast and prostate cancers to ~$65,000 for head and neck cancers, NHL and leukaemia (Table 2). During the terminal phase 76% of costs were for inpatient care, 3% for ED presentations, 11% for prescription medicines and 10% for other medical services covered by the MBS. Higher costs for leukaemia cases were driven by inpatient hospitalisations (83% of costs), including repeated blood/platelet transfusions, and also for head and neck cancers (87% of costs), often including a tracheotomy.

### Prevalence costs

There were 124,465 newly diagnosed cancers in Australia in 2013, with similar numbers each year back to 2009 (117,288 cancers diagnosed). The excess health system costs in 2013 for all people living up to 5 years after a cancer diagnosis were estimated at $6.3billion (Table 3). This represents 6.3% of all government healthcare expenditure in Australia for that year, 0.4% of GDP and equates to $272 per person in Australia. Total costs were highest for colorectal cancers ($1.1billion) and breast cancers ($0.8billion), accounting for 30% of overall costs.

**Table 3:** Total excess costs incurred in 2013 by cancer type for all cases diagnosed in Australia 2009–2013 who were alive in 2013, in millions of Australian dollars.

Cancer type	0–1 year post-diag.	>1–2 years post-diag.	>2–3 years post-diag.	>3–4 years post-diag.	>4–5 years post-diag.	Total
Prostate	349	58	46	41	35	529
Breast	593	115	43	43	31	825
Colorectal	727	137	86	71	45	1,066
Melanoma	91	27	31	27	18	194
Lung	425	77	34	13	7	557
NHL	230	49	26	17	8	330
Head & neck	142	16	-3	4	3	163
Leukaemia	160	71	45	31	35	342
Kidney	110	24	23	17	20	194
Pancreas	129	16	4	0	4	153
Other	1,269	287	179	116	89	1,940
**Total ($mil)**	**4,225**	**878**	**515**	**380**	**295**	**6,293**

diag.: diagnosis; NHL: Non-Hodgkin lymphoma.

For cases who survived at least 5 years, the mean annual excess costs for years 3–5 after diagnosis consistently averaged ~$2,500 per case. In early 2013, there were approximately 1 million Australians living with cancer. The estimated $6.3billion prevalence cost was based on ~480,000 cases—excluding these gives another ~520,000 in the continuing care phase. Applying an annual excess cost of $2,500 per case would give an additional ongoing health system cost of ~$1.3billion per year, although some of these costs would reduce after 10–15 years if a patient is considered ‘cured’ [23] and others would have higher costs as they would be in the terminal phase.

## Discussion

This is the first study in Australia to report population-wide costs of cancer care using individual-level data for all cancers. By calculating excess costs relative to matched controls, we could reliably estimate cancer-specific health system expenditure by cancer type, time since diagnosis and phase of care. Health system costs varied widely by these factors and we have described these variations in considerable detail. We presented the costs by source (hospitalisations, prescription medicines, other medical services and ED presentations) and examined specific healthcare such as surgical procedures and types of medicine. We included the full range of costs of cancer care, from pre-diagnosis (including general practitioner visits, specialists, imaging), through initial treatment, continuing care, and end-of-life care. The costs were dominated by initial treatment and end-of-life care, however continuing care costs for cancer survivors (e.g. ongoing surveillance, medicines) were also substantial.

Our estimated $6.3billion for the direct health services cost of cancer care in 2013 is similar to but slightly higher than the most recent report of cancer-specific expenditure in Australia [6]. This might be due in part to the AIHW analysis being unable to attribute certain costs to cancer care without individual-level information identifying cancer cases. The results are also in line with the NSW report giving “lifetime” costs, which were equivalent to ~$5billion in health system costs for cases diagnosed in Australia in 2005 [7]. Previous studies of costs of end-of-life care for cancer cases reported similar trajectories for costs rapidly increasing in the final months of life [14, 15, 31]. The results are also comparable to a study of cancer costs in all 27 European Union nations that estimated costs in 2009 of €102 per person [32]–our estimate (~€165) would place Australia between Finland (€151, third highest) and Germany (€182, second highest). Other studies that informed the model for our study had similar cost patterns by cancer type and phase of care [21–23].

There are several limitations to this study. The cancer cases may not be representative of all cases in Australia, as the 45 and Up Study had a relatively low response rate and participants have been shown to be healthier and more likely to have private health insurance than the general population [24]. This could lead to higher costs for both cancer cases and cancer-free controls as they might have greater health-seeking behaviour. However, we matched cases with controls within the cohort to minimise the effect of any potential selection bias and the cohort has been shown to give representative estimates of relative differences [33]. As described above, our results were similar to other studies. Disease stage at diagnosis is not recorded for all of Australia so we weighted our sample by stage to match the NSW distribution; however NSW accounts for one-third of all incident cancers in Australia and cancer patterns in NSW are similar to those nationally. All persons in the study cohort were aged > = 45 years so costs for younger cases could not be estimated, although this will have a limited impact on our estimates since cancers occurring in people aged under 45 years comprise only 8.5% of incident cancers in Australia. While we matched cancer-free controls on age and place of residence, the oversampling of people aged > = 80 and those from rural areas in the 45 and Up cohort may impact our cost estimates if these demographic factors have an effect on costs.

There were relatively small numbers of cases by stage for less common cancers, which could lead to imprecise estimates when weighted to population-level distributions. However, the weights used were all within a reasonable range and we were able to match 97% of cases to three controls to reduce the variability in our estimates. Including only cases with three matched controls also made little difference to the results. Relatively small case numbers might give unstable cost estimates for rare cancer types when further stratified by phase of care. However, major findings for the overall cost of cancer and the stratified costs for the major cancer types are likely to be stable, since they were based on large numbers of individuals (e.g. colorectal cancer costs were based on analysis of data for 967 cases and 2870 controls). We did identify negative excess costs prior to diagnosis for prostate and breast cancers but this is likely explained by the demographic characteristics of those who are more commonly diagnosed with these cancers and the potential for increased early detection of these cancer types in those who access health services more often. Negative costs for cases were also possible as controls were not limited to those who were alive throughout the study period, so the controls may have experienced the terminal care phase with the associated higher costs. Only selecting potentially healthier controls who did not die would introduce bias. Our outcome is excess costs for cases relative to their peers so all ranges of health status of the controls were included. Furthermore, some of the cancer cases would have died from non-cancer causes and as they were not excluded, similar controls were not excluded either.

Only month and year of diagnosis were available so the annual excess costs for the 12 months “after” diagnosis started on the first day of the month of diagnosis. This might include some costs associated with diagnosis, particularly for those diagnosed late in the calendar month. However these costs are still attributable to cancer and should not be disregarded, although care is required if apportioning costs between the diagnostic period and post-diagnosis care. Pleasingly, it appears that the full date of diagnosis will be available in future releases of these cancer registry data.

Other methods of selecting comparison groups have been proposed, such as using cases’ pre-diagnosis costs as the “control” costs. However this has been shown to result in higher estimated excess costs compared with the selection of contemporaneous controls based on demographic factors, while the inclusion of health-related factors in matching (such as smoking status in our study) has been shown to improve the accuracy of excess cost estimates [34].

Our estimates of prevalence costs were based on estimated case numbers using historical incidence and survival rather than following all individuals diagnosed in 2009–2013. However the overall number of cases was commensurate with the 5-year prevalence reported for 2012 [5], and the costs were dominated by the first year after diagnosis where we have the actual case numbers, so the impact of applying survival rates over multiple years is limited. A sensitivity analysis using 10% lower survival rates reduced the prevalence costs by a modest 3%, to $6.1billion. We did not include the excess costs in the year prior to diagnosis in our estimate of prevalence costs; if this were to be included, based on the number of new cancers diagnosed in 2013 this would add ~$200million to our prevalent cost estimate.

There are other limitations related to the use of these routinely collected data. For example, we could not account for chemotherapy or radiotherapy delivered in public hospitals on an outpatient basis. Not all EDs in NSW were captured in the EDDC during the study period; in 2007 the EDDC captured over 80% of ED presentations in NSW [35] and by the end of the study period the EDDC had near complete coverage. However ED costs accounted for a very small proportion of healthcare costs, generally less than 2%. Restricting the analysis to geographical areas with complete ED coverage made little difference to the results. Most health services costs are included and the strength of our study design meant there was no reliance on patients’ recall. Ten percent of all cases had a record of healthcare coverage by the Australian Government Department of Veterans’ Affairs. These cases can have their prescription medicines subsidised under a separate billing system whose data were not available for this study. Excluding these cases increased the mean excess costs by around 1–2% for all reported patient groups.

Overall, our estimates are likely to underestimate the total health services costs of cancer care in Australia for the reasons detailed above. Furthermore, in the few years since our analysis period ended, a range of new targeted therapies and immunotherapies have been added to the PBS. This includes pembrolizumab for melanoma, nivolumab for lung cancer and ibrutinib for leukaemia. In future years, other similar medicines are likely to be added to the PBS and some medicines included in the PBS may extend their use to other patient groups, potentially paired with tests for eligibility for these therapies being covered in the MBS. This is expected to have driven up overall costs of cancer care substantially over the last few years, and will also impact the relative ranking of the costs by cancer type. Also, we were unable to report on the costs for non-melanoma skin cancer, or the recently announced increases in Australian government funding for chemotherapy and palliative care. Therefore, we plan time-extended analyses of these costs once further follow-up data on the included cancer cases, and more recently diagnosed incident cases become available in the 45 and Up Study cohort.

We restricted our analysis to direct health system costs due to a lack of comprehensive data for other costs, such as patients’ out-of-pocket costs, personal insurance premiums or expenditure covered by private health insurance. It has previously been estimated that in Australia only 29% of the lifetime financial cost of cancer is borne by the health system, with the main cost component (58%) attributable to carer and productivity costs [7]. Thus, the societal cost of cancer is likely to be significantly more than that estimated here; and if the prior estimate of the fraction of total costs attributable to health services is assumed to apply, our findings imply that the overall societal costs of cancer are of the order of $25 billion per year.

This study has several other key strengths. The findings presented here are likely to be more accurate, inclusive and evidence-based than previously reported estimates and are very important for understanding the drivers of healthcare expenditure. We used a large population-based sample with detailed individual-level data, included all cancer types, adjusted results to reflect the actual population distribution of cancer cases and matched cases to multiple cancer-free controls to give more robust results. The cases were identified through a comprehensive high quality state-wide cancer registry. Previous studies have described the strengths of using cancer registry data for this type of research and the usefulness of administrative health datasets for costing studies [36–38].

Future research with these data could include analyses of indirect costs such as productivity costs, costs borne by carers and burden of disease. Others have been able to report on costs by disease stage where the data were available [21], however detailed breakdown by stage was beyond the scope of this study. Our future work will focus on individual cancer types, with a more detailed description of costs by various patient and tumour characteristics, including disease stage. Further research could also include projections of cancer prevalence by phase of care to estimate future costs [39, 40]. The results can potentially be used to evaluate the cost-effectiveness of changes in cancer care such as the introduction of new drugs, screening programs or primary prevention.

## Conclusions

The excess costs of cancer care are significant and vary substantially by cancer type and time since diagnosis. This is the first Australian study to provide detailed individual-level data for all cancers. The results provide important information for health services planning, implementation and delivery, and the evaluation of potential new interventions in cancer control, allowing more efficient allocation of health resources for the care of people with cancer. These findings emphasise the economic importance of effective primary and secondary cancer prevention strategies.

## Supporting information

S1 File Supporting information, including Figures A-C and Tables A-C. (DOCX)

## References

[1] AIHW. Cancer incidence projections: Australia, 2011 to 2020. Australian Institute of Health and Welfare Canberra, Australia; 2012.

[2] Siddiqui M, Rajkumar SV. The high cost of cancer drugs and what we can do about it. Mayo Clin Proc. 2012;87(10):935–43. 10.1016/j.mayocp.2012.07.007 23036669 PMC3538397

[3] Smith TJ, Hillner BE. Bending the cost curve in cancer care. New Engl J Med. 2011;364(21):2060–5. 10.1056/NEJMsb1013826 21612477 PMC4042405

[4] Karikios DJ, Schofield D, Salkeld G, Mann KP, Trotman J, Stockler MR. Rising cost of anticancer drugs in Australia. Intern Med J. 2014;44(5):458–63. 10.1111/imj.12399 24612257

[5] AIHW. Cancer in Australia 2017. Australian Institute of Health and Welfare Canberra, Australia; 2017.

[6] AIHW. Health system expenditure on cancer and other neoplasms in Australia: 2008–09 Cancer series no. 81. Australian Institute of Health and Welfare Canberra, Australia; 2013.

[7] Access Economics. Cost of Cancer in NSW: A report for the Cancer Council NSW. Sydney, Australia; 2007.

[8] Lew JB, Howard K, Gertig D, Smith M, Clements M, Nickson C, et al Expenditure and resource utilisation for cervical screening in Australia. BMC Health Serv Res. 2012;12:446 10.1186/1472-6963-12-446 23216968 PMC3548768

[9] Cronin P, Kirkbride B, Bang A, Parkinson B, Smith D, Haywood P. Long-term health care costs for patients with prostate cancer: a population-wide longitudinal study in New South Wales, Australia. Asia Pac J Clin Oncol. 2017;13(3):160–71. 10.1111/ajco.12582 27619777

[10] Kang S, Koh ES, Vinod SK, Jalaludin B. Cost analysis of lung cancer management in South Western Sydney. J Med Imaging Radiat Oncol. 2012;56(2):235–41. 10.1111/j.1754-9485.2012.02354.x 22498199

[11] Doran CM, Ling R, Byrnes J, Crane M, Searles A, Perez D, et al Estimating the economic costs of skin cancer in New South Wales, Australia. BMC Public Health. 2015;15:952 10.1186/s12889-015-2267-3 26400024 PMC4581089

[12] Ananda S, Kosmider S, Tran B, Field K, Jones I, Skinner I, et al The rapidly escalating cost of treating colorectal cancer in Australia. Asia Pac J Clin Oncol. 2016;12(1):33–40. 10.1111/ajco.12350 25866889

[13] Ward RL, Laaksonen MA, van Gool K, Pearson SA, Daniels B, Bastick P, et al Cost of cancer care for patients undergoing chemotherapy: The Elements of Cancer Care study. Asia Pac J Clin Oncol. 2015;11(2):178–86. 10.1111/ajco.12354 25865926

[14] Langton JM, Reeve R, Srasuebkul P, Haas M, Viney R, Currow D, et al Health service use and costs in the last 6 months of life in elderly decedents with a history of cancer: a comprehensive analysis from a health payer perspective. British Journal of Cancer. 2016;114(11):1293–302. 10.1038/bjc.2016.75 27115468 PMC4891509

[15] Kardamanidis K, Lim K, Da Cunha C, Taylor LK, Jorm LR. Hospital costs of older people in New South Wales in the last year of life. Med J Aust. 2007;187(7):383–6. 17907999 10.5694/j.1326-5377.2007.tb01306.x

[16] Paul C, Fradgley E, Roach D, Baird H. Impact of financial costs of cancer on patients—the Australian experience. Cancer Forum. 2017;41(2).

[17] Girgis A, Lambert S. Cost of informal caregiving in cancer care. Cancer Forum. 2017;41(2).

[18] Gordon LG, Merollini K, Lowe A, Chan RJ. Financial toxicity—what it is and how to measure it. Cancer Forum. 2017;41(2).

[19] Schubert C. Regulatory and government funding agency consideration of monetary costs to the cancer patient. Cancer Forum. 2017;41(2).

[20] Shih S, Carter R. Measurement of resource utilisation in cancer clinical studies—tools, issues and challenges. Cancer Forum. 2017;41(2).

[21] Laudicella M, Walsh B, Burns E, Smith PC. Cost of care for cancer patients in England: evidence from population-based patient-level data. Br J Cancer. 2016;114(11):1286–92. 10.1038/bjc.2016.77 27070711 PMC4891510

[22] Mariotto AB, Robin Yabroff K, Shao Y, Feuer EJ, Brown ML. Projections of the cost of cancer care in the United States: 2010–2020. Journal of the National Cancer Institute. 2011;103(2):117–28. 10.1093/jnci/djq495 21228314 PMC3107566

[23] Blakely T, Atkinson J, Kvizhinadze G, Wilson N, Davies A, Clarke P. Patterns of cancer care costs in a country with detailed individual data. Med Care. 2015;53(4):302–9. 10.1097/MLR.0000000000000330 25749656 PMC4379114

[24] Banks E, Redman S, Jorm L, Armstrong B, Bauman A, Beard J, et al Cohort profile: the 45 and Up Study. Int J Epidemiol. 2008;37(5):941–7. 10.1093/ije/dym184 17881411 PMC2557061

[25] Kelman CW, Bass AJ, Holman CD. Research use of linked health data—a best practice protocol. Australian and New Zealand journal of public health. 2002;26(3):251–5. 12141621 10.1111/j.1467-842x.2002.tb00682.x

[26] Duckett SJ. Drug Policy Down Under: Australia's Pharmaceutical Benefits Scheme. Health Care Financing Review. 2004;25(3):55–67. 15229996 PMC4194861

[27] Independent Hospital Pricing Authority. National Hospital Cost Data Collection Australian Public Hospitals Cost Report 2010–2011, Round 15. Sydney, Australia; 2012.

[28] Australian Bureau of Statistics. Consumer price index: September Quarter 2016. Canberra, Australia; 2016. Report No.: 6401.0.

[29] Cancer Institute NSW. Cancer in New South Wales: Online Statistics Module [Cited 5-Feb-2018]. http://www.statistics.cancerinstitute.org.au/.

[30] AIHW. Cancer in Australia: an overview 2014. Australian Institute of Health and Welfare Canberra, Australia; 2014.

[31] Langton JM, Blanch B, Drew AK, Haas M, Ingham JM, Pearson SA. Retrospective studies of end-of-life resource utilization and costs in cancer care using health administrative data: a systematic review. Palliat Med. 2014;28(10):1167–96. 10.1177/0269216314533813 24866758

[32] Luengo-Fernandez R, Leal J, Gray A, Sullivan R. Economic burden of cancer across the European Union: a population-based cost analysis. Lancet Oncol. 2013;14(12):1165–74. 10.1016/S1470-2045(13)70442-X 24131614

[33] Mealing NM, Banks E, Jorm LR, Steel DG, Clements MS, Rogers KD. Investigation of relative risk estimates from studies of the same population with contrasting response rates and designs. BMC Med Res Methodol. 2010;10:26 10.1186/1471-2288-10-26 20356408 PMC2868856

[34] Chen AB, Li L, Cronin AM, Brooks GA, Kavanagh BD, Schrag D. Estimating Costs of Care Attributable to Cancer: Does the Choice of Comparison Group Matter? Health services research. 2017. 10.1111/1475-6773.12760 PMC6056589 28858372

[35] O'Connell DL, Goldsbury DE, Davidson P, Girgis A, Phillips JL, Piza M, et al Acute hospital-based services utilisation during the last year of life in New South Wales, Australia: methods for a population-based study. BMJ Open. 2014;4(3):e004455 10.1136/bmjopen-2013-004455 24682576 PMC3975743

[36] Riley GF. Administrative and claims records as sources of health care cost data. Med Care. 2009;47(7 Suppl 1):S51–5. 10.1097/MLR.0b013e31819c95aa 19536019

[37] Yabroff KR, Warren JL, Banthin J, Schrag D, Mariotto A, Lawrence W, et al Comparison of approaches for estimating prevalence costs of care for cancer patients: what is the impact of data source? Med Care. 2009;47(7 Suppl 1):S64–9. 10.1097/MLR.0b013e3181a23e25 19536016 PMC2718428

[38] Yabroff KR, Warren JL, Schrag D, Mariotto A, Meekins A, Topor M, et al Comparison of approaches for estimating incidence costs of care for colorectal cancer patients. Med Care. 2009;47(7 Suppl 1):S56–63. 10.1097/MLR.0b013e3181a4f482 19536010

[39] Yu XQ, Luo Q, Smith DP, Clements MS, Patel MI, O'Connell DL. Phase of care prevalence for prostate cancer in New South Wales, Australia: A population-based modelling study. PLoS One. 2017;12(2):e0171013 10.1371/journal.pone.0171013 28178275 PMC5298320

[40] Yu XQ, De Angelis R, Luo Q, Kahn C, Houssami N, O'Connell DL. A population-based study of breast cancer prevalence in Australia: predicting the future health care needs of women living with breast cancer. BMC Cancer. 2014;14:936 10.1186/1471-2407-14-936 25494610 PMC4295409

