# Supplementary material for: Health services costs for cancer care in Australia: Estimates from the 45 and Up Study
Source: PLoS One. 2018 Jul 30;13(7):e0201552. doi: 10.1371/journal.pone.0201552 (PMC6066250; doi:10.1371/journal.pone.0201552)
Supplement: S1 File — (DOCX) [file pone.0201552.s001.docx]

**Health services costs for cancer care in Australia: Estimates from the 45 and Up Study**

**Supporting information**

**Fig A. Data sources and date coverage.**


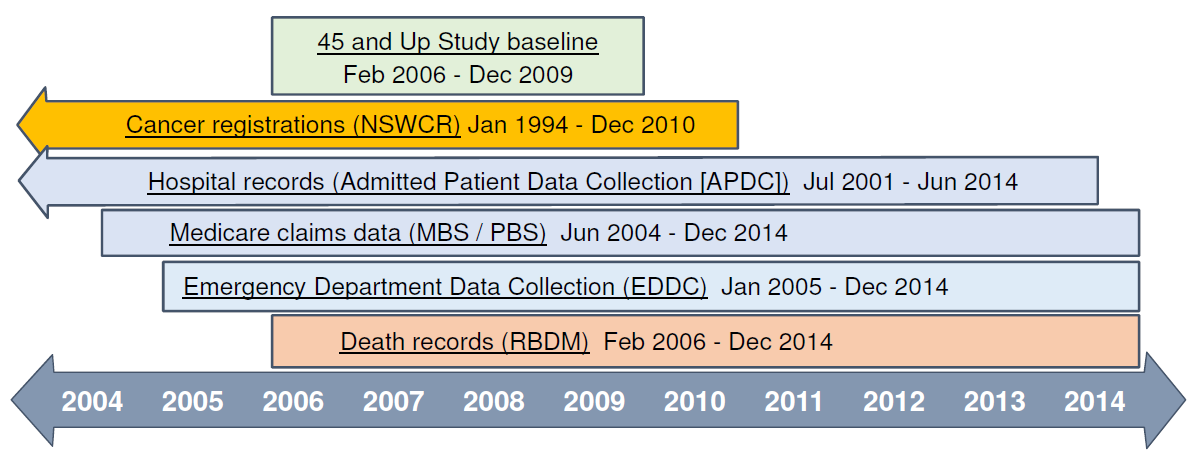


NSWCR: New South Wales Cancer Registry; MBS: Medicare Benefits Schedule; PBS: Pharmaceutical Benefits Scheme; RBDM: Registry of Births, Deaths and Marriages

**Fig B. Mean monthly excess costs for incident cancers diagnosed 2006-2010, relative to the month of diagnosis, for those alive at the start of each month.**

Costs reported as Australian dollars for the year 2013.

**Fig C. Mean monthly excess costs at the end of life for incident cancers diagnosed 2006-2010, by time between diagnosis and death.**

Costs reported as Australian dollars for the year 2013.

**Table A. Distribution of cases included for analysis (diagnosed 2006-2010) compared with cases in the NSW and Australian populations.**

| **Cancer type** | **No. of cases** | **% of cases** | **% of NSW cases 2008-2012** | **% of Australia cases 2013** |
| --- | --- | --- | --- | --- |
| Prostate | 1,944 | 25 | 18 | 15 |
| Breast | 1,010 | 13 | 12 | 13 |
| Colorectal | 967 | 13 | 12 | 12 |
| Melanoma | 891 | 12 | 10 | 10 |
| Lung | 523 | 7 | 9 | 9 |
| NHL | 261 | 3 | 4 | 4 |
| Head & neck | 143 | 2 | 3 | 3 |
| Leukaemia | 143 | 2 | 3 | 3 |
| Kidney | 141 | 2 | 3 | 2 |
| Pancreas | 157 | 2 | 2 | 2 |
| Other | 1,444 | 19 | 24 | 26 |
| **Total** | **7,624** | **100** | **100** | **100** |

NHL: Non-Hodgkin lymphoma; NSW: New South Wales

**Table B. Summary statistics for annual excess costs for incident cancers diagnosed 2006-2010, for those alive at the start of each time period*.**

|  | **>1-2 years**  **pre-diag.** | **>0-1 year**  **pre-diag.** | **0-1 year**  **post-diag.** | **>1-2 years**  **post-diag.** | **>2-3 years**  **post-diag.** | **>3-4 years**  **post-diag.** | **>4-5 years**  **post-diag.** |
| --- | --- | --- | --- | --- | --- | --- | --- |
| Mean | $314 | $1,622 | $33,944 | $8,796 | $5,624 | $4,454 | $3,643 |
| Standard deviation | $12,432 | $14,626 | $37,635 | $24,090 | $21,205 | $20,399 | $19,233 |
| Median | -$614 | -$114 | $24,076 | $1,839 | $547 | $171 | -$83 |
| Quartile 1 | -$3,490 | -$3,081 | $10,142 | -$1,728 | -$2,757 | -$3,214 | -$3,995 |
| Quartile 3 | $2,125 | $3,587 | $47,353 | $12,039 | $7,293 | $5,949 | $5,317 |

* Weighted to the distribution of cancer types in Australia in 2013 and the New South Wales stage distribution. Costs reported as Australian dollars for the year 2013.

**Table C. Summary statistics for excess costs by phase of care for incident cancers diagnosed 2006-2010*.**

|  | **Phase of care** | | |
| --- | --- | --- | --- |
|  | **Initial** | **Continuing (p.a.)** | **Terminal** |
| Mean | $28,719 | $4,474 | $49,733 |
| Standard deviation | $32,151 | $17,390 | $49,861 |
| Median | $20,703 | $1,203 | $37,750 |
| Quartile 1 | $7,277 | -$2,663 | $18,577 |
| Quartile 3 | $40,629 | $7,698 | $68,159 |

* Weighted to the distribution of cancer types in Australia in 2013 and the New South Wales stage distribution; p.a.: per annum. Costs reported as Australian dollars for the year 2013.

**Other notes**

**Cancer types**

Specific cancer types were identified using the NSWCR topography code, based on the International Classification of Diseases and Related Health Problems, Tenth Revision, Australian Modification (ICD-10-AM): prostate (C61), breast (C50), colorectal (C18-C20), melanoma (C43), lung (C33-C34), non-Hodgkin lymphoma (NHL; C82-C85), head and neck (C01-C14,C30-C32), leukaemia (C91-C95), kidney (C64), pancreas (C25) and all others combined (“other”).

**Costs by phase of care**

The mean excess cost during the continuing phase for head and neck cancers was negative, suggesting these cases require less healthcare than controls during this period. However the median excess cost per case was $1184 per year and three of the matched controls had extremely high costs. Removing these ‘case groups’ from the estimates raised the mean excess costs for cases to $547 per year.
